# Effects of P_4_ Antagonist RU486 on VEGF and Its Receptors’ Signaling during the In Vivo Transition from the Preovulatory to Periovulatory Phase of Ovarian Follicles

**DOI:** 10.3390/ijms222413520

**Published:** 2021-12-16

**Authors:** Annunziata Mauro, Paolo Berardinelli, Valentina Russo, Nicola Bernabò, Alessandra Martelli, Delia Nardinocchi, Oriana Di Giacinto, Maura Turriani, Barbara Barboni

**Affiliations:** 1 Faculty of Bioscience and Technology for Food, Agriculture and Environment, University of Teramo, Via R. Balzarini 1, 64100 Teramo, Italy; pberardinelli@unite.it (P.B.); vrusso@unite.it (V.R.); nbernabo@unite.it (N.B.); amartelli@unite.it (A.M.); dnardinocchi@unite.it (D.N.); odigiacinto@unite.it (O.D.G.); mturriani@unite.it (M.T.); bbarboni@unite.it (B.B.); 2 Institute of Biochemistry and Cell Biology (IBBC), National Research Council, A. Buzzati-Traverso Campus, Via E. Ramarini 32, Monterotondo Scalo, 00015 Rome, Italy

**Keywords:** gonadotropins, progesterone, signal transduction, ERKs, AKT, ovary, preovulatory follicle, periovulatory follicle, angiogenesis, reproduction

## Abstract

The development of an adequate blood vessel network is crucial for the accomplishment of ovarian follicle growth and ovulation, which is necessary to support the proliferative and endocrine functions of the follicular cells. Although the Vascular Endothelial Growth Factor (VEGF) through gonadotropins guides ovarian angiogenesis, the role exerted by the switch on of Progesterone (P_4_) during the periovulatory phase remains to be clarified. The present research aimed to investigate in vivo VEGF-mediated mechanisms by inducing the development of periovulatory follicles using a pharmacologically validated synchronization treatment carried out in presence or absence of P_4_ receptor antagonist RU486. Spatio-temporal expression profiles of VEGF, FLT1, and FLK1 receptors and the two major MAPK/ERKs and PI3K/AKT downstream pathways were analyzed on granulosa and on theca compartment. For the first time, the results demonstrated that in vivo administration of P_4_ antagonist RU486 inhibits follicular VEGF receptors’ signaling mainly acting on the theca layer by downregulating the activation of ERKs and AKTs. Under the effect of RU486, periovulatory follicles’ microarchitecture did not move towards the periovulatory stage. The present evidence provides new insights on P_4_ in vivo biological effects in driving vascular and tissue remodeling during the periovulatory phase.

## 1. Introduction

Physiological and pathological processes involve blood vessel remodeling such as vasculogenesis, angiogenesis, and formation and maintenance of blood vessel structures. To date, they have been largely studied in embryo/foetal development [1], tissue growth and wound healing [2], ovarian cell cycle [3,4,5,6,7,8,9,10,11,12], placenta development [13] tumorigenesis and cancer [14,15,16,17], rheumatoid arthritis [18], diabetic retinopathy [19,20], axon growth [21,22], and inflammation [23].

The key process of blood vessel remodeling recognizes common mechanisms involving inhibitory and stimulatory growth factors, and amongst the former ones basic fibroblast growth factor (bFGF) [24] and vascular endothelial growth factor (VEGF) are considered to be the major ones.

The female gonad is one of the most studied examples of adult tissue where cyclically active angiogenesis alternates phases of blood vessel network quiescence or regression. At each reproductive cycle, dominant preovulatory follicles are selected from a pool of grown antral follicles. This process leads to ovulation, first, and to cyclic or gestational corpus luteum (CL) formation later [25,26]. The development of a specific blood vessel network is required for the success of the process of follicle dominance. Indeed, the ovarian blood vessel network is crucial to sustain the enhanced proliferative and endocrine functions of the preovulatory follicles and to create the angiogenetic basis for CL development [6,7,25,26,27]. Blood vessels support growing follicles in acquiring an exponentially increasing amount of nutrients and molecules, essential as precursors for the synthesis of steroids and other regulating ovarian hormones released into systemic circulation [6,28,29,30,31]. Several key factors are recognized as follicle angiogenesis drivers and they have been indirectly traced in controlling this final reproductive outcome [6,7,10,11,30,31,32,33,34,35,36,37]. VEGF is recognized to be one of the most relevant angiogenetic factors studied to date in orchestrating folliculogenesis [8,28,36]. Indeed, VEGF increased secretion has been documented for the transition from large preantral to early antral follicles [3,31,38,39]. Conversely, the process of follicle atresia is characterized by an early sign of VEGF decrease then followed by the reduction of the blood vessel network [12,34,40,41,42]. In an opposite way, VEGF is up regulated in dominant follicle/s by triggering mechanisms leading to ovulation [4,10,38,43]. Luteinizing hormone (LH) surge, during a short window lasting from 24 to 44 h depending on the species, is responsible to trigger the profound morphological and functional changes occurring during the transition, leading a preovulatory follicle to become periovulatory before it ovulates [42,44]. These events, in parallel, recognize a dynamic remodeling of blood vessels network through a LH-mediated VEGF pathway [5,7,36]. More in detail, LH surge induces a progressive disorganization of follicle basal membrane caused by activation of proteolytic enzymes [33,36]. Furthermore, LH changes the steroid enzymatic pathway converting an estrogen secreting structure, the preovulatory follicle, into a P4-producing one (periovulatory follicle) by activating a process of follicle functional luteinization before ovulation and morphological CL formation [45,46]. During this phase, follicular blood vessels undergo to dramatic modifications. Large blood vessels appear for the first time in follicle walls and with a higher blood flow [5,10,11,30,47]. Experimental evidences have shown that this vascular remodeling is controlled by VEGF [5,6,30,31,38,43]. Indeed, VEGF inhibition reduces endothelial cell (ECs) proliferation and hinders follicle angiogenesis, which, in turn, prevents periovulatory follicle ovulation [8,9,43,48]. Even if the role of VEGF on follicle development is clearly established, the mechanisms involved in its local modulation remain to be clarified.

It is known that VEGF exerts its angiogenic effects, in particular on ECs, by binding to two high-affinity tyrosine kinase receptors, FLT1 (VEGF receptor (1) and FLK1 (VEGF receptor (2) [49,50]. Ligand binding to FLT1 induces autophosphorylation on multiple intracellular tyrosine residues leading different downstream signal transduction pathways such as PI3K, Nck, and SHP-2 [49,50,51]. FLT1 has a very high affinity for VEGF but a relatively low activity of protein kinase may act as a negative regulator of VEGF signaling by limiting the amount of free VEGF and preventing its binding to FLK1, the major receptor of VEGF, and plays a chief role in angiogenesis [52,53]. The VEGF binding to FLK1 induces the receptor dimerization, activation, and trans-auto-phosphorylation of the tyrosine kinase, and then the phosphorylation molecules of intracellular signaling cascades, such as PKC- Raf-MEK-ERKs, PI3K-Akt, SHB-FAK-paxillin, SHB-PI3K-Akt, and NCK-p38-MAPKAPK2/3 pathways. Downstream intracellular signaling targets enter the nucleus and, by binding to transcription factors, induce multiple-gene expression in response to extracellular stimuli [52,53]. Amongst them, it has been also reported that ERKs and PI3K/AKt signaling pathways, in particular, play crucial roles in the development and steroidogenesis of the ovarian follicle [54,55,56].

Even if LH surge seems to link the final follicle specialization (periovulatory phase and ovulation) and blood vessel remodeling through VEGF expression [4,5,10,11,33,57,58,59], it remains also to clarify whether gonadotropins directly influence VEGF expression or, on the contrary, if the angiogenic factor is indirectly influenced by the follicular steroidogenesis, as suggested by in vitro [57,60] and in vivo experiments [61].

It has been reported that the in vitro LH stimulatory effects on ERK1/2 phosphorylation and in granulosa cells from 9- to 14-mm follicles were abolished by specific chemical inhibition of VEGF receptor 2 (VEGFR2), suggesting important roles of VEGF and its VEGFR2 receptor in mediating and/or enhancing the effects of gonadotropins in granulosa cells [62].

P_4_ could have a function in inducing VEGF expression [63,64], endothelial cell proliferation [65,66], and angiogenesis [3,67]. In fact, the P_4_ antagonist molecule, RU486, administration significantly reduced the in vivo synthesis of VEGF in rat ovary [68,69], in monkey endometrium [63], and in pig ovary [6]. In particular, it has been demonstrated that RU486 strongly downregulates theca VEGF expression in swine species, thus preventing the angiogenic response in almost 70% of the ovarian follicles which did not achieve the characteristic periovulatory organization. Indeed, RU486-treated follicles exhibited a lower rate of endothelial cell proliferation, a reduced recruitment of perivascular mural cells, and a reduced vascular area, providing significant insights on the steroid hormone’s biological role [6]. These results also confirm that the morphological and vascular remodeling recognize controlling cross-talk mechanisms [67,70,71,72,73,74].

Even if P_4_ effects ovarian blood flow [38,75,76,77] during the periovulatory stage, when the LH surge induces a complete inversion of follicular steroid secretion [28,78], the role exerted by the steroids on follicular VEGF/receptors family synthesis, as well as the signal transduction pathways activated, still remains to be investigated.

To this aim, the present in vivo research was designed to study the mechanisms mediating the VEGF activated pathways after a validated, inductive ovulatory pharmacological treatment [5,6,10,11,31]. In particular, the expression profile of VEGF’s receptors and their major MAPK/ERKs and PI3K/AKT transduction signaling were analyzed in gilts pre (before hCG) and periovulatory follicle walls at 36 h from hCG administration. In addition, in order to determine whether VEGF’s signaling cascade is influenced by P_4_, the inductive ovulatory treatment was performed in the presence of P_4_ receptor antagonist RU486 [79,80,81,82]. The morphological and biochemical analysis of VEGF-mediating signaling was assessed and compared between the two experimental conditions (pre and periovulatory ovarian follicles’ transition induced in the presence or absence of RU486).

## 2. Results

### 2.1. Morphological and Functional Ovarian Response to Hormonal Treatment

**Figure 1:**
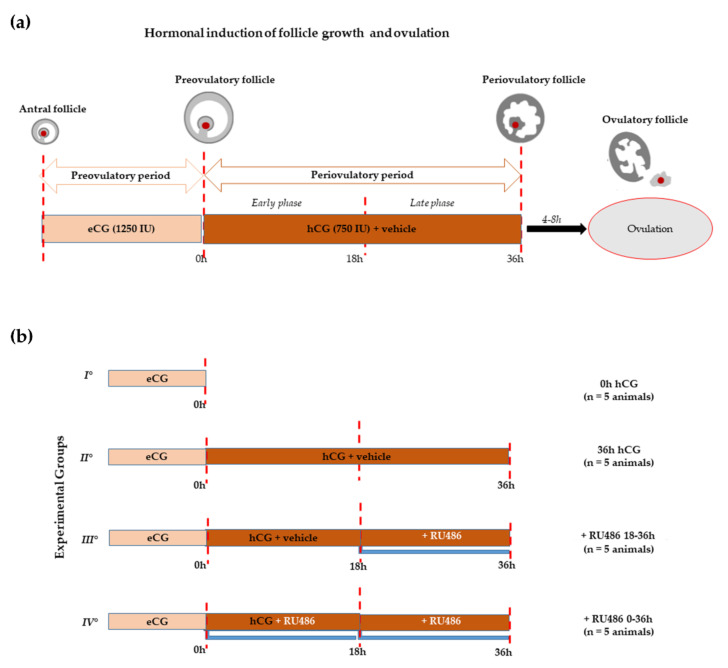
Experimental design. (**a**) Schematic illustration of in vivo hormonal treatment of ovarian synchronization of prepubertal gilts able to promote antral follicle recruitment (preovulatory phase) using eCG and the induction of ovulation by hCG (periovulatory phase). The periovulatory phase was stopped a few hours before ovulation (36 h after hCG instead of 40–44 h). (**b**) Scheme of treatments: all groups of animals (n = 5 each) received the superovulatory treatment of 1250 IU eCG. Sixty-two hours later, the first group (I°) was ovariectomized to obtain preovulatory follicles (0 h hCG), while the remaining groups received the ovulatory treatment consisting of 750 IU hCG with or without RU486, both dissolved in corn oil vehicle. The second group (II°) includes animals ovariectomized 36 h later (36 h hCG). The third group (III°) of animals received hCG and, 18 h later, a single dose of RU486 injection (+RU486 18–36 h). Finally, the fourth group (IV°) was treated with hCG in combination with a double dose of RU486, performed at the moment- of hCG injection and 18 h later (+RU486 0–36 h).

The follicles isolated from ovaries of prepubertal gilts exposed to hormonal treatment of ovarian synchronization (Figure 1, Experimental design) showed that RU486 did not macroscopically affect the periovulatory phase.

More in detail, the number of preovulatory follicles (diameter > 6 mm) selected after 62 h from a mild superovulatory protocol (1250 IU eCG or 0 h hCG; Figure 1a), adopted to avoid any unphysiological ovarian response, was similar to all the recruited ovarian follicles into the perivulatory phase triggered through the ovulatory treatment of 750 IU hCG (Figure 1a): 20.98 ± 4.05 (0 h hCG) vs. 21.026 ± 1.74 (36 h hCG), respectively (*p* > 0.05, Table 1). The periovulatory transition induced by hCG in the presence RU486 injection did not affect follicle recruitment. Indeed, 18.966 ± 1.62 and 19.026 ± 1.74 periovulatory follicles were recorded in gilts treated with double doses of RU486 (+RU486 0–36 h; Figure 1b) in order to cover the whole periovulatory phase with the treatments, or with a single dose carried out 18 h after hCG, in order to modulate the late luteinization window (+RU486 18–36 h; Figure 1b), respectively (Table 1). At the same time, RU486 treatment did not impact the periovulatory follicle’s diameter, as summarized in Table 1.

**Figure 2:**
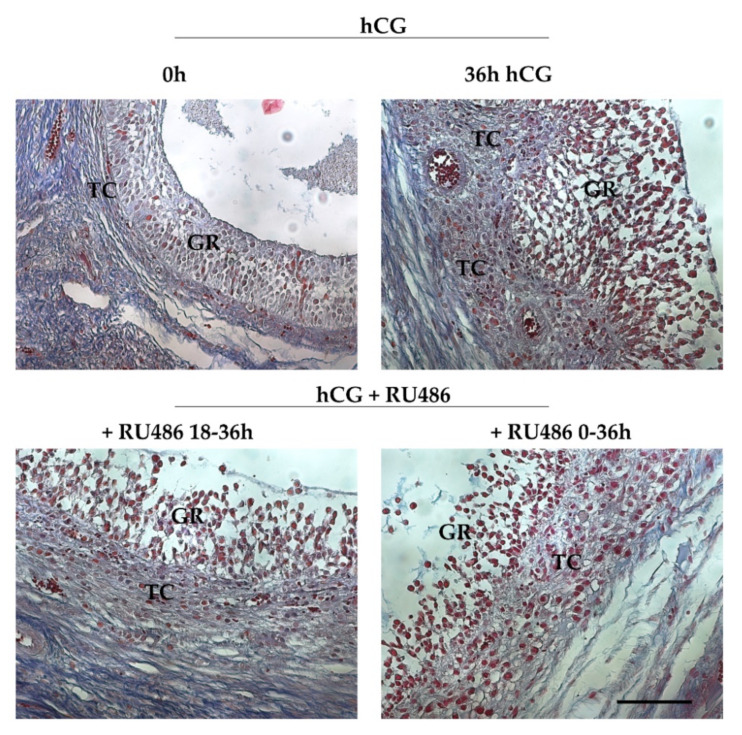
Periovulatory ovarian follicles’ morphology induced by RU486 treatment. Representative images stained with Azan Mallory showing the microarchitecture of preovulatory (0 h hCG) and periovulatory follicles (36 h hCG, +RU486 18–36 h and +RU486 0–36 h). The images of periovulatory follicles isolated after RU486 treatments displayed an altered periovulatory TC and GR compartment organization. Scale bar: 100 μm. Abbreviations: GR, granulosa layer; TC, theca layer.

RU486-treatment induced some morphological differences into periovulatory follicles’ microarchitecture. Indeed, Azan-Mallory staining (Figure 2) showed that all the pre and periovulatory follicles isolated were healthy with the oocytes surrounded by a continuous cumulus cell layer. The germ cells displayed a uniform cytoplasm and a GV with a condensed peri-nucleolar chromatin (data not shown). Preovulatory follicles (0 h hCG, Figure 2) showed a spherical shape with a compact mural granulosa (GR) and theca (TC) layers apposed on an intact basal membrane. Differently, periovulatory follicles isolated 36 h after hCG (36 h hCG, Figure 2) displayed TC compartment projecting into the GR and its cells showed clear signs of expansion. Differently, the majority of RU486-treated follicles (from 75% to 84%) maintained a preovulatory TC and GR compartment organization except for the expansion of GR cells, independently of the drug period injection (Figure 2).

RU486 administration, in addition, induced significant differences in follicular fluid (FF) VEGF content. More in detail, the FF of preovulatory follicles (0 h hCG) had high levels of VEGF (13.966 ± 0.53 ng/mL) that dropped, becoming undetectable (n.d.) in periovulatory structures after 36 h from hCG administration. Of note, RU486 treatment, even if it induced a dramatic decrease in VEGF FF content, maintained its level on mean values of 0.956 ± 0.07 and 0.986 ± 0.07 ng/m in 18–36 h and 0–36 h RU486 treatment, respectively (Table 2).

### 2.2. hCG Administration Modulates Ovarian Follicle VEGF-Related Pathway in GC and TC Follicular Compartments

**Figure 3:**
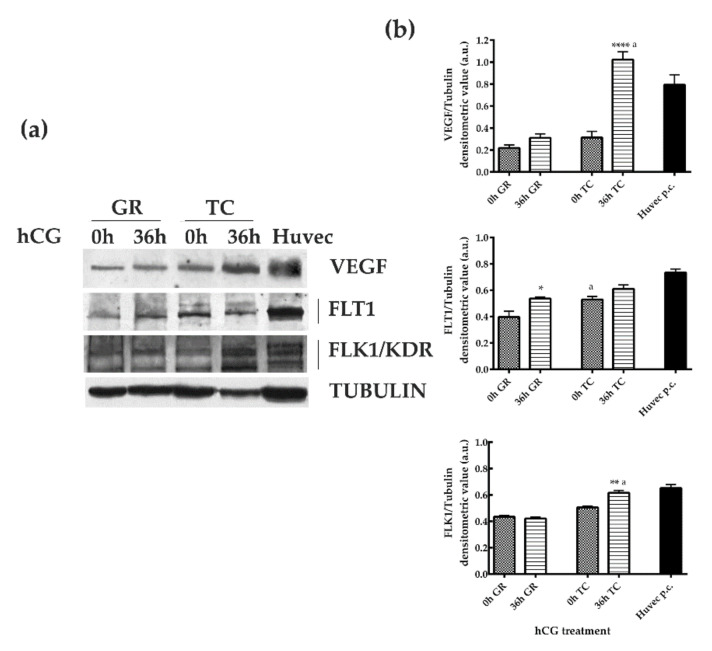
VEGF and FLT1/VEGFR1 and FLK1/VEGRF2 receptors’ expression in GR and TC compartments during the transition from pre- to periovulatory phase. (**a**) Representative Western Blot (WB) images of VEGF, FLT1, and FLK1 protein carried out on equal amounts (75 μg) of protein extracted from GR or TC compartement of large follicles isolated during the preovulatory (0 h hCG) and periovulatory phase (36 h hCG). Huvec cells were used as positive control sample for proteins expression. (**b**) The histograms indicate relative densitometric values normalized on Tubulin expression. The values are expressed as mean ± S.D. of three independent experiments. Statistically different values for: * *p* < 0.05, ** *p* < 0.005, **** *p* < 0.0001 vs. 0 h hCG, ^a^ *p* < 0.05 vs each time treatment calculated within each follicular compartment (GR or TC).

VEGF protein levels (Figure 3) were similar in GR and TC compartments of preovulatory follicles (0 h hCG). The protein content did not change in GR after hCG treatment (0 h vs. 36 h hCG, *p* > 0.05), while it increased in TC compartment (0 h vs. 36 h hCG *p* < 0.05). However, the VEGF levels of the TC layer remained significantly higher than those of GR compartment at the end of the periovulatory phase (GR 36 h hCG vs. TC 36 h hCG, *p* < 0.0001).

The analysis of FLT1 and FLK1/KDR protein expression confirmed the compartment and temporal receptors’ modulation (Figure 3) promoted by hCG treatment.

In detail, the levels of FLT1 significantly increased in the GR compartment close to ovulation (0 h hCG vs. 36 h hCG, *p* < 0.05), whereas it maintained constant levels in TC (0 h hCG vs. 36 h hCG, *p* > 0.05). Moreover, FLT1 were present at lower levels in GR compartment of preovulatory follicles (GR 0 h hCG vs. TC 0 h hCG, *p* < 0.05), and then reached similar values between GC and TC layers in periovulatory structures (GR 36 h hCG vs. TC 36 h hCG, *p* > 0.05; Figure 3). In an opposite way, FLK1 showed a constant trend of expression in GR during the transition from the pre to periovulatory phase (0 h CG vs. 36 h hCG, *p* > 0.05) and then increased its levels in the TC layer 36 h later hCG treatment (0 h hCG vs. 36 h hCG *p* < 0.05). Overall, the FLK1 levels became significantly higher in the TC layer of periovulatory follicles (GR 36 h hCG vs. TC 36 h hCG, *p* < 0.05; Figure 3).

### 2.3. P_4_ Receptor Antagonist RU486 Modulated VEGF Related Pathways in Granulosa and Theca Compartments

**Figure 4:**
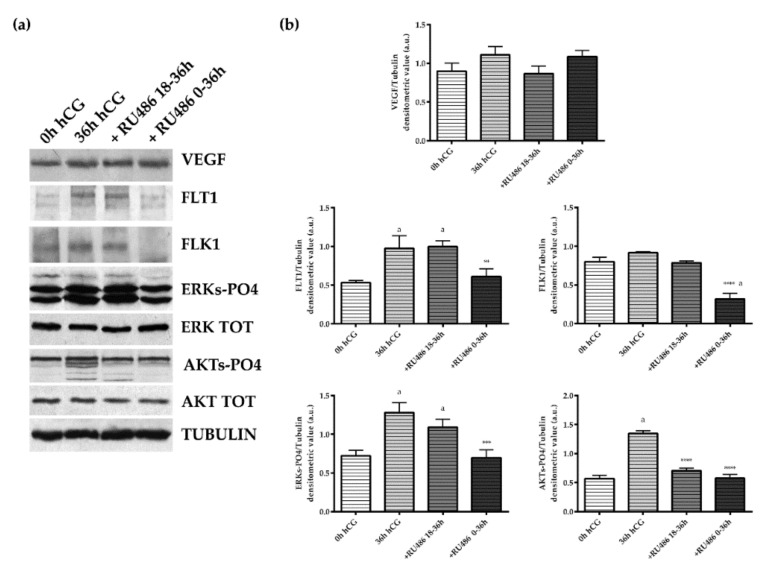
VEGF, receptors, and downstream ERKs and AKTs phosphorylation patterns detected in the GR of pre and periovulatory follicles derived from animal treated with or without the P_4_ antagonist, RU486. (**a**) Representative WB images of VEGF, FLT1, FLk1, ERKs-PO4, total ERK, AKT-PO4, and total AKT protein expressions carried out on equal amounts (50 μg) of total protein extracted from GR of pre and periovulatory follicles obtained from animals before (0 h hCG) or 36 h after hCG (36 h hCG) in the presence or absence of RU486 (+RU486 18–36 h or +RU486 0–36 h). (**b**) Representative densitometric values of proteins normalized on Tubulin expression. The values are expressed as mean ± S.D. of three independent experiments. Statistically different values for: ** *p* < 0.005, *** *p* < 0.001, and **** *p* < 0.0001 vs. 36 h hCG; ^a^ *p* < 0.05 vs. 0 h hCG.

The levels of VEGF receptors in the GR layer were modulated by RU486 with a greater degree of efficacy when the administration of the antagonist was maintained during whole the periovulatory period by using a double dose (Figure 4). More in detail, RU486 0–36 h significantly reduced the expression of both VEGF receptors in GR layer (Figure 4) without affecting the VEGF levels (Figure 4). In particular, the double dose of RU486 0–36 h was able to prevent the increase in the FLT1 receptor (*p* < 0.05 vs. 36 h hCG) in GR layer and, at the same time, to significantly reduce FLK1 content (*p* < 0.001 vs. 36 h hCG). On the contrary, both the inhibitory influences were not induced when the RU486 treatment was performed with a single dose (+RU486 18–36 h, Figure 4).

**Figure 5:**
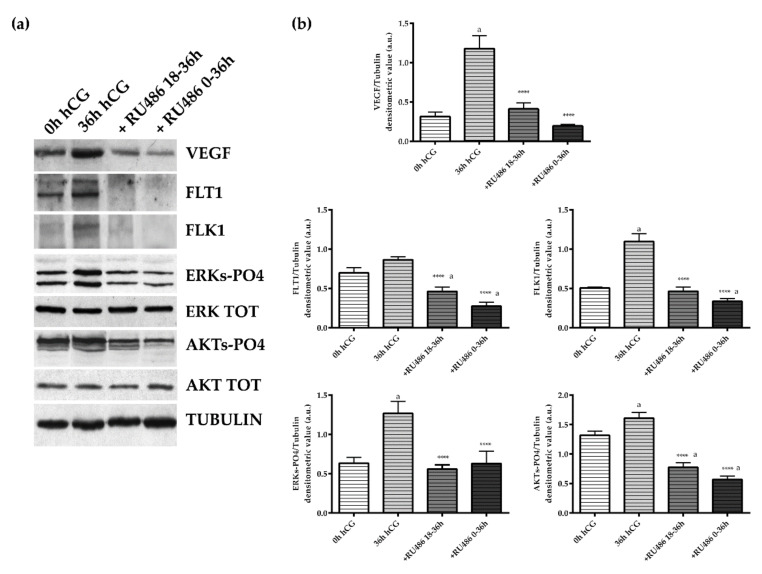
VEGF/receptors and downstream ERKs and AKTs phosphorylation in the theca layer of periovulatory follicles derived from animals treated with P_4_ antagonist RU486. (**a**) Representative WB images of VEGF, FLT1, FLk1, ERKs-PO4, ERK Total, AKT-PO4, and AKT protein expressions carried out on equal amounts (50 μg) of total protein extracted from the TC layer of large periovulatory follicles obtained from animals before hCG (0 h hCG) and 36 h after hCG in the presence of the corn oil vehicle (36 h hCG) or RU486 administered at a later time (+RU486 18–36 h) or for the whole periovulatory period (+RU486 0–36 h). (**b**) Representative proteins densitometric values normalized on Tubulin expression. The values are expressed as mean ± S.D. of three independent experiments. Statistically different values for: **** *p* < 0.0001 vs. hCG; ^a^ *p* < 0.05 vs. 0 h hCG.

In order to better clarify the VEGF-related signaling, two key down-streams of VEGF pathways, MAPK/ERKs and AKTs, were explored. The physiological kinetic of their status of activation during the pre and periovulatory phase was evaluated in both follicular compartments (GR, Figure 4; TC, Figure 5). The analysis showed, first of all, that in the GR compartment of preovulatory follicles (0 h hCG), the status of activation was higher for ERKs (ERKs-PO4, refers to both phosphorylated ERK 1 and ERK 2) than for AKT (AKT-PO4) (Figure 4). Moreover, both pathways were dramatically activated during the periovulatory phase (36 h hCG). Indeed, the phosphorylated isoforms of both ERKs and AKTs displayed in the GR layer 3.9- and 2.4-fold increased, respectively (for both kinase 0 h hCG vs. 36 h hCG *p* < 0.0001, Figure 4), despite similar levels of respective total proteins (semiquantitative data not shown in the histogram). Of note, a coherent modulation of VEGF key downstream kinases was recorded in the GR compartment after RU486 0–36 h treatment where both the activations of ERKs and AKTs were strongly inhibited (+RU486 0–36 h vs. 36 h hCG *p* < 0.001, for both kinases). In particular, both the levels of ERKs- and AKTs-phosphorylated isoforms were similar to those recorded in hCG pre-treatment (+RU486 0–36 h vs. 0 h hCG, *p* > 0.05). Conversely, the exposure to RU486 during the late window of periovulatory phase did not impact on the status of GR ERKs activation, whereas it significantly inhibited the AKTs degree of phosphorylation (+RU486 18–36 h vs. 36 h hCG *p* < 0.001), reaching concentrations that were similar to those induced by the double dose of P_4_ antagonist (+RU486 0–36 h vs. +RU486 18–36 h, *p* > 0.05).

The TC compartment appeared more sensible to RU486 exposure. Indeed, the antagonist administration was able to affect the VEGF pathway during the whole periovulatory phase (Figure 5). Indeed, both single and double RU486 doses (18–36 h and 0–36 h) prevented VEGF accumulation in TC compartment (single and double RU486 doses vs. 36 h hCG *p* < 0.0001). A similar inhibitory influence was observed on the expression of FLT1 and FLK1 receptors. Both receptors became almost undetectable in periovulatory follicles collected after 18–36 h and 0–36 h RU486 injection (both RU486 treatments vs. 36 h hCG *p* < 0.0001 for FLT1 and FLK1, respectively; Figure 5). Conversely to the GR compartment (Figure 4), the TC layer displayed a status of AKT activation (AKT-PO4) higher than ERKs (ERKs-PO4) (0 h hCG, Figure 5). More in detail, in TC the AKT-PO4 levels were 2.3-fold higher in preovulatory follicles (GR 0 h hCG vs. TC 0 h hCG, *p* < 0.01, Figure 4 and Figure 5). Nevertheless, the levels of activations of both phosphorylated isoforms of ERKs and AKTs increased in periovulatory phase reaching 1.5- and 1.3-fold higher levels, respectively (for both kinase 0 h hCG vs. 36 h hCG *p* < 0.0001, Figure 5) 36 h after hCG treatment. Coherent with the inhibited expression of VEGF and related receptors, a reduced status of phosphorylation of ERKs and AKT were recorded after RU486 treatments (RU486 treatments vs. 36 h hCG, *p* < 0.0001; Figure 5). These results suggest a more effective influence of RU486 action on the TC layer, which was evident during both early and late periovulatory periods.

## 3. Discussion

The present study aimed at clarifying the role of P_4_ in controlling ovarian angiogenesis with a spatio-temporal VEGF-mediated mechanism during the periovulatory phase. The current results demonstrated, for the first time, that VEGF receptors and the related downstream ERKs and AKTs pathways were affected by the in vivo administration of the P_4_ antagonist, RU486, during the transition from the pre to periovulatory phase. This evidence confirmed the inhibitory effect of RU486 on the blood vessel network remodeling of pig ovarian follicles that are close to ovulation [6], but most of all start to describe the mechanisms involved. New insights on RU486’s negative effect on reproductive outcomes were reported in this study, thus confirming the previous evidence that suggests a P_4_-mediated mechanism in controlling pregnancy [82,83,84], ovulation [85,86,87,88], and sperm-oocyte recognition [89]. In addition, the data collected in the present research support the idea that RU486 was able to specifically inhibit the VEGF-dependent angiogenetic mechanisms during the periovulatory phase into a compartment and time-dependent manner.

Beyond VEGF [5,6,11], the present results focused on FLT1 and FLK1 receptors and the two major related VEGF pathways, MAPK/ERKs and PI3K/AKTs, the targets of P_4_ action during the transition from the pre to periovulatory phase in pig ovarian follicles.

Interestingly, VEGF downstream signaling was differently modulated in the follicular compartments. Indeed, MAPK/ERKs pathway widely prevailed in GR compartment of preovulatory follicle, whereas PI3K/AKTs was highly operative in the TC layer. The hCG treatment, mimicking the LH surge, strongly activated both pathways. It was interesting to note that both the downstream kinases of VEGF pathway could be antagonized by blocking the P_4_ action with associated profound morphological and functional follicular changes during the periovulatory phase. These differences recorded in main downstream VEGF signaling could open the hypothesis of different roles in each follicular compartment. Numerous studies have already demonstrated the expression of both the VEGF receptors, FLT1 (VEGFR1) and FLK1/KDR (VEGFR2), in GR as well as their dynamic changes throughout the ovarian follicles development in primates [38], pigs [90], cattle [91], and bovine [62]. In this context, independently of its angiogenic effects, VEGF had been positively related to proliferation, [62] survival [92], and differentiation of the follicular response by acting through VEGF-FLK1-ERKs pathways activation both in vitro [62] and in vivo [93]. In particular, it has been demonstrated that VEGF-mediated ERK1/2 phosphorylation mechanisms are involved in the modulation of bovine and mouse GR cells by directly promoting in vitro proliferation [62,92]. Moreover, GR cells exposure to a selective FLK1/KDR (VEGFR2) inhibitor blocked the switch on of steroidogenesis in dominant follicles by preventing ERK1/2 phosphorylation induced by LH [62]. Other studies using ruminant-derived cells have suggested that VEGF may also modulate GR function directly by promoting cell survival [60]. The literature collected to date supports the idea of a role of ERKs in GR cells but without demonstrating any precise phase of activation (preovulatory and periovulatory) under in vivo conditions.

A biphasic effect of VEGF and its related receptors was supposed in GR: VEGF-ERKs activation may exert in preovulatory follicles a preliminary stimulatory effect on cell proliferation to then become differentiative during the periovulatory period [94]. According to the literature, the FLK1 receptor could be the main intracellular transducer of the VEGF signal [52,53] in GR and TC compartments. The FLT1, which is considered a sort of decoy receptor, probably is involved in linking the soluble isoform of the angiogenic factors that physiologically drops (undetectable levels) in the FF during the periovulatory phase. VEGF-FLK1 binding seems to be involved in starting the signal cascade of events leading to downstream ERKs activation able to promptly induce the biological response of GR/TC luteinization in the periovulatory follicle. The evidence collected after in vivo *Erk1/2* GR disruption as well as the short time ERK1/2 activation in preovulatory follicles exposed to LH seems to support the hypothesis of a role of the ERK downstream signal in reprogramming preovulatory GR cells induced by LH/hCG [93]. RU486 treatment did not affect GR and FF VEGF content, but strongly reduced the levels of VEGF in the TC layer, as well as its receptors and both ERKs and AKTs signaling pathways, bringing them to the levels present in the preovulatory follicles and by pointing into a major role of this follicular compartment in driving the morphological transition involving somatic and blood vessel networks towards the periovulatory phase.

However, even if results on reduction on VEGF/receptors and their downstream targets induced by RU486 support the hypothesis of P_4_ in controlling GR and TC periovulatory fate, further regulatory mechanisms cannot be excluded. Indeed, a variety of other stimuli might regulate PI3K/AKT signaling pathways in GR as IGF-1, EGF, and/or physical and mechanical stimuli, including cell adhesion, ovarian tissue density, and Hippo pathways [95]. Moreover, relationships between of ERKs and PI3K/AKT signaling pathways and follicular steroidogenesis are also demonstrated by acting as specific sensing mechanisms of ovarian cells’ nutritional state [54,55,56]. Literature data support the coexistence of the activation of both pathways in both follicular compartments in which, through multiple and complex regulatory mechanisms of interactions, they ensure the viability of the follicle during the transition from the pre to periovulatory stage sustaining ovulation and CL formation. Even if a direct cause effect correlation between RU486 and MAPK/ERKs and/or PI3K/AKT signaling deactivation cannot be demonstrated, however, a clear and coherent pattern of inhibition was induced on VEGF levels and kinases activation in TC compartment.

The inhibitory action exerted by RU486 on VEGF-mediated signaling may also be involved in the dramatic changes previously recorded in TC blood vessel remodeling during the periovulatory stage [6]. This idea is supported by the scientific evidence collected to date demonstrating that VEGF/FLK1, mainly through the activation of TSAd-Src-PI3K-AKT signaling pathways, regulate the survival of human umbilical vein endothelial cells [52,96]. On the other hand, VEGF by FLK1 binding regulates the proliferation of the endothelial cells through the PLCγ-PKC-Raf-MEK-ERKs signaling pathway [52]. The FLK1 receptor was also described as a modulator of the endothelial cells’ migration and vascular permeability [52].

Specifically, RU486 has been described to impair, in TC compartments during the transition from the pre to periovulatory phase, the endothelial cells’ proliferation and migration, leading to the increase and stabilization of the follicular inner and outer blood vessel follicular networks [5]. In this context, the increased availability in the TC of the VEGF-receptor binding may explain the coherent activation of both ERKs and AKT signaling pathways in TC endothelial cells. Even if a direct relationship between P_4_ and periovulatory events cannot be demonstrated, however, the opposite follicular effects promoted by RU486 treatment strongly support this hypothesis. Indeed, RU486 was able to selectively inhibit in the TC layer VEGF content [6] and of its receptors by, in turn, negatively influencing the status of phosphorylation of both ERKs and AKTs triggered by hCG. In parallel, the majority of periovulatory follicles collected from RU486-treated pigs displayed an incomplete development of the blood vessel and reduced extension of the outer network of the vascular area by failing to complete the blood vessel remodeling [6], leading to ovulation [85,86,87,88]) and morphological luteinization. A similar RU486 inhibitory effect has been reported in bovine [97], human [98], and mink [99], and in other reproductive tissues such as the endometrium [63,64,67,100] exposed to low concentrations of P_4_. Although RU486 is classically described as an anti-progestin in reproductive tissue, its anti-glucocorticoid effects [101] cannot be excluded. However, regardless of these caveats about the anti-progesteron or anti-glucorticoid role, robust experimental evidence has demonstrated a role of RU486 is the regulation of ovarian folliculogenesis, inhibition of ovulation, and modulation of luteal function [101]. Starting from this evidence, the present manuscript further investigated the involvement of RU486 in mediating key transduction pathways. Even if the use of the in vivo model did not allow the documentation of a cause-effect correlation between the studied events, the results collected clearly confirmed a strong role of RU486 in preventing hCG-induced ovarian follicle remodeling, in modulating the synthesis of VEGF, and the activation of two major related signaling cascades during the phase of transition from pre to periovulatory follicle. It can only be hypothesized that these RU486 inhibitory effects may be related to the an anti-progesterone action, as reported in bovine [97], human [98], and mink [99], and in endometrium [63,64,67,100] by considering the high level of P_4_ characterizing the phase of transition from the pre to periovulatory follicle [46]. Despite this important evidence, further experiments are still required to confirm the VEGF/receptors’ signaling P4-dependent role in controlling the follicular periovulatory events. In this context, signal transduction specific inhibitors, siRNA molecules, or knockout animal models will be the unique tools to dissect follicle specific events even if this, on in vivo large mammal models, is quite difficult to achieve.

In conclusion, the present results demonstrated that RU486 administration during the periovulatory phase inhibit the VEGF signaling pathways mainly in the TC compartment by contributing into a downregulation of GR and TC ERKs and AKTs activations.

This evidence provides new insights on the biological in vivo role of the P_4_ antagonist in driving vascular and tissue remodeling during the transition from the preovulatory to the periovulatory stage in the pig model by introducing a new role in controlling female reproductive outcomes.

## 4. Materials and Methods

### 4.1. Ethical Committee

The study was carried out according to the Declaration of Helsinki guidelines and approved by the Universities of Teramo and Chieti-Pescara Ethics Committee (Prot. 81/2011/CEISA/COM). Surgery of the animals was performed under anesthesia with sodium pentobarbital. Eventual suffering was minimized with all efforts.

### 4.2. Animal Treatments and Ovary Collection

In 20 prepubertal Large White gilts with a mean weight of 90.761 Kg (mean ± S.D.), follicular growth and ovulation were induced pharmacologically using two sequentially intramuscular (i.m.) administrations of 1250 IU of eCG (Folligon; Intervet International B.V.-AN Boxmeer, Netherlands) and 750 IU of hCG (Corulon; Intervet International-Boxmeer, AN Boxmeer, Netherlands), respectively [5,6,10,11,31] (Figure 1a). The animals were divided into five experimental groups as summarized in Figure 1b. Ovariectomies [5,6] were performed in order to collect preovulatory follicles 62 h after eCG treatment (0 h hCG, I° group; n = 5 animals), while the periovulatory ones were collected 36 h after hCG injection dissolved in corn oil vehicle (36 h hCG, II° group; n = 5 animals) (Figure 1b), which does not modify the hormone action as previously reported in Mauro et al. [6]. In order to investigate the RU486’s (Mifepristone, M8046, Sigma-Aldrich, St. Louis, MO, USA) [6,80,81] effects on periovulatory follicles, the hCG injection was conducted in combination with RU486 at different time points during the periovulatory period (Figure 1b). In particular, RU486 was solubilized in corn oil (10 mL) and i.m. injected at a concentration of 10 mg/kg as previously validated [6,80]. The double dose of RU486 was planned by considering the drug half-life span, which is about 18–20 h [102]. To this aim: (i) one RU486 administration was carried out 18 h after hCG, during the late phase of the periovulatory period (+RU486 18–36 h, III° group, n = 5 animals), and (ii) two consecutive RU486 administrations were carried out for the whole periovulatory period, the first one in combination with hCG, the second one 18 h later, in corn oil (+RU486 0–36 h, IV° group; n = 5 animal) (Figure 1b). The hCG solubilized in corn oil represents the experimental control (hCG+vehicle, experimental CTR) (Figure 1b).

### 4.3. Sample Preparation

After performing the ovariectomy, one ovary was fixed in 4% paraformaldehyde/phosphate-buffered saline (PBS; pH 7.4) for 12 h at 4 °C, dehydrated, and embedded in paraffin wax for histology. The contra lateral ovary was harvested, as previously reported [5,6,11], to isolate each healthy preovulatory (8 mm ≤ Ø ≥ 7 mm) and periovulatory follicle (11 mm ≤ Ø ≥ 8 mm). Under a stereomicroscope, each single follicle was opened to obtain the FF and isolate the follicular wall. The dissection of each follicular wall was carried out to separate the granulosa cells from the theca shell [5,6,10,11]. In detail, each structure was transferred into the dissection medium and the granulosa layer was carefully scraped away, by using a small spatula, from the theca shell. The dispersed granulosa cells contained in the medium were collected and centrifuged. Instead, the theca shell was vigorously vortexed and washed to avoid granulosa cell contamination [6,11]. Samples of FF, granulosa, and theca were individually stored in liquid nitrogen.

### 4.4. Morphological and Morphometric Analyses of the Follicular Response

Paraffin tissue sections, five μm in thickness, were serially collected on poly-L-lysine-coated slides, according to previous reports [5,6], before been analyzed in terms of morphology and morphometry. In particular, a series of 20 sections at a distance of 100 μm/ovary was used. Tissue sections were stained with haematoxylin–eosin (HE) and Azan-Mallory to identify healthy follicles and to assess their development stage. According to previous reports [5,6], only healthy large antral follicles (diameter > 6 mm) were analyzed. Follicles were considered as healthy if they enclosed an oocyte with a normal-shape and delimited by granulosa cells with normal appearance of their nuclei (without signs of pyknosis) regularly apposed on an intact basal membrane. If follicles did not fulfill the abovementioned criteria, they were considered unsuitable for analysis. The follicular diameter was measured using the computed image analysis system (KS300; Zeiss) which allowed us to measure two diameters of the follicle sections perpendicularly and then the mean diameter was calculated from these measurements. Then, for each morphometric analysis, at least two sections/follicle were randomly chosen and the whole cross-follicular wall was analyzed. Morphometric analyses were carried out at × 400 Magnification with an Axioscop-2plus epifluorescence microscope (Axioskop 2 Plus, Carl Zeiss, Oberkochen, Germany) equipped with KS300, an interactive and automatic image analyzer (AxioVision, Version 3.1, Carl Zeiss, Oberkochen, Germany).

### 4.5. VEGF Content in FF

A specific ELISA assay (Human VEGF Quantikine ELISA Kit; R&D Systems, Minneapolis, MN, Canada, USA) was used to analyze VEGF content in FF according to previous reports [5,6,11]. At least five different samples of FF/hormonal treatment were used to quantify VEGF soluble levels, which were expressed as ng/mL (mean values ± S.D.). The limited amounts of FF did not allow us to assay other molecules.

### 4.6. Protein Content in Granulosa and Theca Compartments and Western Blot Analyses

Total proteins extracted from granulosa or theca layers of each single isolated follicle (n = 5 follicles/gilt/treatment) were processed according to Mauro et al. [6]. Proteins (50–75 ug) were separated by 8%–12% SDS-PAGE and electrophoretically transferred into a nitrocellulose membrane (Whatman Protran Nitrocellulose Membrane, Merck-Millipore, Bedford, MA, USA) with Trans-Blot TURBO Transfer System (Bio-Rad Laboratories, Hercules, CA, USA). Membranes were subsequently incubated with blocking solution of 5% not-fat-dried milk (Sigma) in buffer containing 0.1% (*v*/*v*) Tween 20 in Tris HCl pH 7,5-buffer saline (T-TBS) for 1 h at RT. Protein detection was performed by incubating the membranes with the primary antibodies in Table 3. The goat anti-rabbit or goat anti-mouse immunoglobulins peroxidase-conjugated (IgG-HRP) were finally used as secondary antibody (Table 3). Target proteins were visualized by ECL substrate (LiteAblot Plus, Euroclone, Milan, Italy) and chemiluminescent signal was detected with Azure’s 400 (Azure Biosystems, c400, Sierra Ct, Dublin, CA, USA). Densitometric analysis were performed with Image J Blot analyzer software (ImageJ 1.53k, NIH, USA, https://imagej.nih.gov). Protein expression semiquantitative data were expressed as the mean ratio of the optical density of specific bands normalized for the Tubulin expression.

### 4.7. Statistical Analysis

The data were expressed as mean ± S.D. of at least 3 independent experiments/treatment. The quantitative data obtained from the different hormonal treatments were firstly assessed for normalcy of distribution by D’Agostino and Pearson test. Then, the data sets were compared using One-way ANOVA test followed, when necessary, by post-hoc Tukey test (GraphPad Prism 8, GraphPad Software, San Diego, CA, USA). The data were considered significant at least for *p* < 0.05.

## Figures and Tables

**Table 1:** Rate of preovulatory and periovulatory follicles collected in different experimental animal groups.

hCG Performed after 60 h from eCG Superovulatory Treatment		
Hormonal Treatments	Number of Follicles	Follicle Mean Diameter (Ø).
0 h hCG *	20.98 ± 4.05	7.46 ± 0.4 mm
36 h hCG	21.04 ± 1.564	9.5 ± 1.9 mm
+ RU486 18–36 h	19.026 ± 1.74	9.6 ± 1.3 mm ^a^
+ RU486 0–36 h	18.966 ± 1.62	9.7 ± 0.4 mm ^a^

* these data were collected at the moment of hCG treatment in order to assess the efficacy of eCG-superovulatory treatment in recruiting preovulatory follicles. All data are expressed as mean ± SD of 5 different animal replicates. ^a^ Statistically different values vs. 36 h hCG group for *p* > 0.05.

**Table 2:** VEGF protein content in follicular fluid (FF) of preovulatory and periovulatory follicles.

Hormonal Treatments	Number of Follicles	VEGF ng/mL (Mean ± SE)
0 h hCG	20.98 ± 4.05	13.966 ± 0.53
36 h hCG	21.04 ± 1.564	n.d ^a^
+RU486 18–36 h	19.026 ± 1.74	0.956 ± 0.07 ^a^
+RU486 0–36 h	18.966 ± 1.62	0.986 ± 0.07 ^a^

n.d.: non detectable values. ^a^ statistically different values (*p* < 0.05) vs. 0 h hCG.

**Table 3:** Antibody details used for Western Blot analyses.

Primary Antibody (Company Information)	Dilution	Secondary Antibody (Company Information)	Dilution
VEGF (Calbiochem- cat. PC37)	1:500	Anti-rabbit HRP conjugated (Santa Cruz Biotec. sc 2357)	1:1000
VEGFR1/FLT1 (Santa Cruz Biotec. Sc-316)	1: 1000	Anti-rabbit HRP conjugated (Santa Cruz Biotec. sc 2357)	1:1000
VEGFR2/FLK1 (Santa Cruz Biotec. Sc-6251)	1: 1000	Anti-mouse HRP conjugated (Santa Cruz Biotec. cc-516102)	1:1000
ERK (Santa Cruz Biotec. sc-154)	1:1000	Anti-mouse HRP conjugated (Santa Cruz Biotec. sc-516102)	1: 2000
phospho-ERK1/2 (E-4) (Santa Cruz Biotec. sc-7383)	1:1000	Anti-mouse HRP conjugated (Santa Cruz Biotec sc 516102)	1:2000
AKT (C67E7) (Cell Sign. Technology, cat 4691)	1:500	Anti-rabbit HRP conjugated (Santa Cruz Biotec. sc 2357)	1:1000
Phospho-AKT(Ser473) (Santa Cruz Biotec, sc-33437)	1:500	Anti-rabbit HRP conjugated (Santa Cruz Biotec. sc 2357)	1:1000
α-Tubulin (Sigma-Aldrich-T5168)	1:1000	Anti-mouse HRP conjugated (Santa Cruz Biotec sc 51610)2	1:3000

## Data Availability

Data supporting reported results can be obtained upon request to the authors.
